# Transcriptional profiling defines dynamics of parasite tissue sequestration during malaria infection

**DOI:** 10.1186/s13073-015-0133-7

**Published:** 2015-02-27

**Authors:** Karell G Pelle, Keunyoung Oh, Kathrin Buchholz, Vagheesh Narasimhan, Regina Joice, Danny A Milner, Nicolas MB Brancucci, Siyuan Ma, Till S Voss, Ken Ketman, Karl B Seydel, Terrie E Taylor, Natasha S Barteneva, Curtis Huttenhower, Matthias Marti

**Affiliations:** Department of Immunology and Infectious Diseases, Harvard School of Public Health, Boston, MA 02115 USA; Department of Biostatistics, Harvard School of Public Health, Boston, MA 02115 USA; Department of Pathology, Brigham and Women’s Hospital, Boston, MA 02115 USA; Swiss Tropical and Public Health Institute, 4051 Basel, Switzerland; Program in Cellular and Molecular Medicine, Children’s Hospital, Boston, MA 02115 USA; College of Osteopathic Medicine, Michigan State University, East Lansing, MI 48825 USA; Blantyre Malaria Project, University of Malawi College of Medicine, Blantyre, 3 Malawi; Department of Pediatrics, Harvard Medical School, Boston, MA 02115 USA; The Broad Institute of Harvard and MIT, Cambridge, MA 02142 USA

## Abstract

**Background:**

During intra-erythrocytic development, late asexually replicating *Plasmodium falciparum* parasites sequester from peripheral circulation. This facilitates chronic infection and is linked to severe disease and organ-specific pathology including cerebral and placental malaria. Immature gametocytes - sexual stage precursor cells - likewise disappear from circulation. Recent work has demonstrated that these sexual stage parasites are located in the hematopoietic system of the bone marrow before mature gametocytes are released into the bloodstream to facilitate mosquito transmission. However, as sequestration occurs only *in vivo* and not during *in vitro* culture, the mechanisms by which it is regulated and enacted (particularly by the gametocyte stage) remain poorly understood.

**Results:**

We generated the most comprehensive *P. falciparum* functional gene network to date by integrating global transcriptional data from a large set of asexual and sexual *in vitro* samples, patient-derived *in vivo* samples, and a new set of *in vitro* samples profiling sexual commitment. We defined more than 250 functional modules (clusters) of genes that are co-expressed primarily during the intra-erythrocytic parasite cycle, including 35 during sexual commitment and gametocyte development. Comparing the *in vivo* and *in vitro* datasets allowed us, for the first time, to map the time point of asexual parasite sequestration in patients to 22 hours post-invasion, confirming previous *in vitro* observations on the dynamics of host cell modification and cytoadherence. Moreover, we were able to define the properties of gametocyte sequestration, demonstrating the presence of two circulating gametocyte populations: gametocyte rings between 0 and approximately 30 hours post-invasion and mature gametocytes after around 7 days post-invasion.

**Conclusions:**

This study provides a bioinformatics resource for the functional elucidation of parasite life cycle dynamics and specifically demonstrates the presence of the gametocyte ring stages in circulation, adding significantly to our understanding of the dynamics of gametocyte sequestration *in vivo*.

**Electronic supplementary material:**

The online version of this article (doi:10.1186/s13073-015-0133-7) contains supplementary material, which is available to authorized users.

## Background

Malaria remains a major human health problem despite intense efforts to control the disease and to reduce parasite burden globally. The most virulent human malaria parasite, *Plasmodium falciparum*, causes approximately 600,000 deaths each year, mostly among children in sub-Saharan Africa [1]. Malaria parasites have a complex life cycle that includes an obligate switch between the vertebrate host and the mosquito vector. Disease is the result of parasite amplification within red blood cells (RBCs), causing pathology such as anemia and strong inflammatory responses due to the release of parasite antigens into circulation and contact-dependent activation of endothelial cells. During human infection, parasitized erythrocytes predominantly contain asexual cells, and there is only a small fraction of parasites progressing to the sexual stages that are transmissible by mosquitoes. The molecular mechanisms by which *P. falciparum* regulates the rate of sexual conversion have been difficult to characterize globally due to their uniquely host-specific nature and the corresponding lack of good *in vitro* or animal model systems.

Late asexually replicating parasite stages sequester away from the bloodstream deep in host tissues, and this process is linked to organ-specific pathology such as cerebral malaria and pregnancy-associated disease. Tissue sequestration requires large-scale remodeling of the host RBC during early asexual parasite development [2,3], and it is mediated by specific variantly expressed parasite antigens that, once exported to the infected RBC surface, interact with receptors on endothelial cells [4]. This variegated expression of surface antigens is a hallmark of protozoan parasites, including *Plasmodium*, and results in a ‘variantome’ of genes whose expression may greatly differ among infected patients. In *P. falciparum*, the *var* gene family encodes different variants of the exported erythrocyte membrane protein 1 (PfEMP1). Acting as a major cytoadherence determinant, PfEMP1 is also a prime target of humoral immune responses [5]. In order to minimize exposure to the host immune system and at the same time maintain its adherence properties, expression of the protein is epigenetically regulated such that only one copy of the encoding *var* gene repertoire is active per parasite at a given time, while the remaining approximately 60 variants are transcriptionally silent. Likewise, a number of other putative virulence gene families display a variant expression pattern in order to maintain propagation of the parasite in the context of host diversity and immune pressure [6,7]. These include *rifin*, *stevor*, *Pfmc-2TM*, *phist*, *fikk* kinases and acyl-CoA synthases, as well as a subset of parasite ligand genes required for host cell invasion (for example, [8,9]). Genome-wide analyses of epigenetic marks demonstrated that these gene families are regulated by tri-methylation of lysine 9 at the amino-terminal tails of histone H3 (H3K9m^3^) [10,11], a conserved modification that confers variegated gene expression in many eukaryotic organisms [12]. Recently, Rovira-Graells and colleagues [13] investigated transcriptional variation across clones derived from a common parent population and found overlap between variantly expressed genes and the presence of H3K9m^3^ marks.

During each replication cycle, a small subset of asexual parasites becomes committed to produce gametocytes. These sexual cells do not contribute to pathology but are essential for the progression of the life cycle to the mosquito vector [14]. Recently, a transcriptional master regulator, AP2-G, was identified to be required for gametocyte formation in both *P. falciparum* and the rodent malaria parasite *Plasmodium berghei* [15,16]. Reminiscent of virulence gene control, *ap2-g* transcription and the concomitant switch from asexual proliferation to gametocyte production is epigenetically regulated through H3K9m^3^ [17,18]. In *P. falciparum*, gametocyte development progresses through five morphologically distinct stages (stages I to V). After 8 to 12 days of maturation, male and female forms circulate in the bloodstream ready to be transmitted to a mosquito vector. By contrast, immature cells are absent from blood circulation. These developing gametocytes sequester in the hematopoietic system of the human bone marrow instead [19]. Since only the mature gametocyte stages are transmissible, understanding the mechanisms by which parasites initiate sexual differentiation and sequestration provides a promising new target for transmission intervention strategies.

Global transcriptional profiling approaches have provided valuable information on the dynamics of gene expression in malaria parasites, typically by assessing the maintenance of asexual replication *in vitro*. These efforts demonstrated that gene expression occurs as a continuous cascade, with the transcription of most genes reaching a maximum only once per intra-erythrocytic developmental cycle (IDC) [20], and translation being delayed by approximately 10 hours [21]. *Plasmodium* spp. display a striking paucity of conserved sequence-specific transcriptional regulators. The parasite, however, encodes an expanded family of plant-like transcription factors and these ApiAP2 proteins, including AP2-G, have emerged as key players in the regulation of cell cycle progression [22]. In addition, a series of histone modifications are involved in coordinating expression during asexual development [10,11]. The resulting co-expression patterns have allowed the inference of functional gene networks across the IDC, both in the presence or absence of drug perturbations [23,24]. Such studies have defined and validated both conserved and *Plasmodium*-specific clusters of co-expressed genes during the asexual parasite cycle, the latter being involved in processes such as host cell invasion or remodeling.

These studies, as well as the majority of global transcriptional analyses published so far, are based on data gained from *in vitro* parasite cultures and show only minimal differences across distinct parasite isolate strains. However, there is increasing evidence that *in vitro* conditions only capture a fraction of the transcriptional plasticity of the parasite exhibited during *in vivo* infection. For example, a study on uncomplicated malaria patients in Senegal has demonstrated the presence of different physiological parasite states during the IDC, which have not been previously observed under *in vitro* conditions [25]. More recently, transcriptional analysis of cerebral malaria patients in Malawi identified two transcriptional clusters with opposite correlations to parasitemia [26]. Additionally, a comparative analysis between the transcriptomes of clinical isolates and culture-adapted lines suggests differential expression of multiple genes across the RBC parasite cycle [27]. These include genes important for pathogenesis, such as the *var* genes, which show 100-fold down-regulation during culture adaptation [28].

The goal of this study was to generate and characterize a comprehensive functional gene network in *P. falciparum*, incorporating a large number of *in vivo* parasite transcriptional profiles from malaria patients as well as previously analyzed *in vitro* time courses. We have also included a new set of transcriptional profiles from the onset of gametocyte development. We identified over 250 co-expressed functional modules (clusters) within this integrated network, comprising both asexual regulatory programs and gametocyte-specific processes. This allowed us to determine the temporal dynamics of gene expression during asexual and sexual development in human infection and the variability of functional module expression across patients. Further, by comparing this *in vivo* data to *in vitro* time course information, we gained insights into the sequestration dynamics of both asexual and sexual parasites within the host.

## Methods

The research described below conformed to the Helsinki Declaration.

### Ethics statement

This study was approved by the institutional review boards of the Harvard School of Public Health, Brigham and Women’s Hospital and the University of Malawi College of Medicine. Consent was obtained from the patient or a child’s guardian.

### Functional network construction

#### Input datasets and co-expression analysis

Processed data from three *in vivo* datasets [25,26,29] and six *in vitro* time course datasets [20,30-32] were obtained from PlasmoDB (version 10.0) and first filtered to exclude paralogs of the highly polymorphic *var*, *rifin*, and *stevor* gene families to minimize hybridization bias based on sequence variation across parasite strains. Individual genes not present in more than half of the *in vitro* time course studies or more than half of the *in vivo* studies were also removed. Each sample of all datasets was then separately normalized into z-scores using the Sleipnir tool Normalizer [33]. Per-dataset co-expression networks were computed by calculating all pairwise Pearson correlations within each dataset, then Fisher transforming and z-scoring all values [34]. The resulting networks for *in vivo* (field samples) and *in vitro* (time courses) matrices were next combined separately by average z-score meta-analysis [35]. This process averages the normalized correlation values (edges) from within each individual dataset to produce one network each for *in vivo* and *in vitro* data. Pairwise co-expression values (edges) missing between genes G1 and G2 in one of these two networks (as a result of genes not present in individual datasets) were k nearest neighbor imputed by identifying the most heavily weighted 10 neighbors of G1, extracting their connection weights with G2, identifying the closest neighbors of G2, extracting their connection weights with G1, and averaging the extracted weights. Finally these two networks were averaged to provide a global *Plasmodium* co-expression network that equally weighted *in vitro* and *in vivo* transcriptional activity. All correlation calculations and network manipulation were performed using the Sleipnir software package [33].

### Network clustering and functional module definition

The global functional network was used as an input similarity measure for agglomerative hierarchical clustering using complete linkage. Since the network defines an edge weight (normalized co-expression) between all gene pairs, this provides a more nuanced replacement for, for example, Pearson correlation or Euclidean distance as a clustering similarity measure among genes. The resulting gene tree was cut at the 40th percentile of all gene-to-gene normalized co-expression values to identify tightly linked clusters. Clusters with fewer than five genes were excluded from further analysis, and the remaining clusters numbered arbitrarily for subsequent convenient reference (Additional files 1 and 2).

### Functional enrichment analysis and annotation of clusters

Fisher’s exact test was used to annotate each cluster with significant enrichments for a variety of external gene sets (Additional files 1 and 2); in each case, annotation significance was determined by Benjamini-Hochberg false discovery rate (FDR) correction for multiple hypothesis testing over all clusters. Each cluster’s overlap was compared with: i) Gene Ontology (GO) [36] terms, as provided by Bioconductor package org.plasmo.db in R; ii) predicted exported proteins (‘exportome’) as defined by Sargeant *et al.* [37]; iii) host cell invasion proteins based on the presence of ‘invasion’ in the gene product description on PlasmoDB; iv) stage-specific gene expression for gametocytes as defined by Joice *et al.* [29]; v) sexual commitment (this study, see below); iv) differential expression in HP1 knock-down parasites compared with wild-type control [18]; vi) co-expression with *PFL1085w* (this study, see below); vii) variant expression across field strains (this study, see below); viii) variant expression across *in vitro*-adapted parasites (‘variantome’) [13]; ix) the presence of H3K9m^3^ histone marks as defined previously [11,38]; x) differential expression between *in vitro* and field samples (this study, see below).

Finally, clusters were also tested for enrichment of genes associated with clinical phenotypes [26]. To control for the effect of stage on phenotypes, we first assigned patient samples to early (<15 hours) versus late (≥15 hours) stage groups. Each clinical variable’s residuals after regressing on stage group were used as the phenotype’s values adjusting for stage [26]. For each phenotype residual-gene pair, a one-sided *P*-value was calculated; this was either a Kruskal-Wallis test for discrete phenotypes or a Fisher transformed Spearman correlation for continuous phenotypes. These were then aggregated per cluster by combining the *P*-values of all phenotype-gene pairs within the cluster using Simes method. Benjamini-Hochberg FDR correction was again applied to adjust for multiple comparisons.

### Clusters up-regulated in HP1 knock-down versus wild type

We used a linear mixed effects model to identify genes with differential expression in HP1 knock-down parasites versus wild type in the published dataset from the original study [18]. The linear model was fit by assuming each cluster had constant expression within each of three intervals (0 to 6 hours, 7 to 9 hours, and 10 to 12 hours), and that each gene in the cluster was a random effect expressed with Gaussian error around a cluster-specific mean. Coefficients corresponding to 7 to 9 hours and 10 to 12 hours of each cluster were transformed to form two uncorrelated z-scores. *P*-values were defined as normal density greater than mean of two z-scores and adjusted using Benjamini-Hochberg FDR to form adjusted q-values. All clusters with adjusted q-values <0.05 were then defined as up-regulated clusters in HP1 knock-down compared with wild type. These data are shown in Additional file 3.

### Variant expression across *in vivo* samples

Genes, and thus clusters, were differentiated into those constitutively up-regulated (expressed), down-regulated (under-expressed), variant, or none of the above across patient samples. Constitutively expressed genes were defined as those within the top 5% rank sum across the entire transcriptome in all of the three *in vivo* datasets. Constitutively unexpressed genes were similarly defined as those within the bottom 10%. Variantly expressed genes in each field sample were defined as those with variance greater than the 20th percentile across genes within each dataset, excluding those constitutively expressed and constitutively unexpressed.

### Gene and cluster peak times across asexual and sexual cycles

We calculated asexual and sexual peak expression times for individual genes and, in their aggregate, overall for each cluster. For the former, the tight asexual 52-hour time course of the 3D7 reference strain as published by Bozdech *et al.* [20] was used to analyze asexually enriched, commitment, and gametocyte ring clusters. A cubic smoothing spline with five degrees of freedom was fitted to the time course data for each gene. Fit of the model was tested using F-test with 5, *n* - 5 degrees of freedom where *n* represents the total number of non-missing time points. After adjusting for multiple comparisons by using Benjamini-Hochberg FDR, genes with adjusted q-values >0.05 were deemed to have no specific peak signal and not assigned a peak time. For the remaining genes, peak time was defined as the hour within the 52-hour time course at which the smoothed spline achieved the maximum value.

To determine peak times of sexual stage gametocyte genes (excluding those in commitment clusters as described above), the NF54 time course published by Young *et al.* [30] was used. The 13-day time course was divided into at most three segments for each gene where a linear model was fitted at each segment. The number of segments and the end-points of segments were identified based on scanning all combinations of segments (1, 2, or 3) and all possible cutoffs and choosing the combination minimizing total mean squared error. Based on the fit of linear models within the resulting segment(s), peak time was defined as the day within the 13-day time course at which the fitted value achieved the maximum. Asexual and sexual peak times were calculated using these two different models due to the smaller number of gametocyte time points available (13 instead of 52), which precluded fitting the more detailed spline model to the latter dataset.

### Differential gene expression between *in vivo* and *in vitro* samples

*In vivo* samples were compared to the *in vitro* datasets to test for *in vivo* up- or down-regulation of each cluster. Within each field or *in vitro* sample, gene expression was separately standardized to z-scores. Next, for each cluster’s set of genes within each dataset (*in vivo* or *in vitro*), these z-scores were averaged per sample. Finally, for each cluster, a one-sided *t*-test was performed comparing the average z-score vector from the *in vivo* and *in vitro* datasets. Benjamini-Hochberg FDR correction was used to adjust for multiple comparisons across clusters. The same process was used to define differential expression of field samples compared with *in vitro* time courses with all field samples as the reference and for comparison with ring stages with the first 22 hours of three 52-hour time course strains (3D7, DD2, HB3) published previously [20,31] as a reference.

### Patients and sample collection

Patients who enrolled in an ongoing cerebral malaria study [39] at the Queen Elizabeth Central Hospital during the 2010 and 2011 transmission seasons were included in this study. These patients were between the ages of 1 month and 14 years and came from Blantyre, Malawi and surrounding areas, where transmission is high and seasonal. All patients enrolled in the study met the clinical criteria for cerebral malaria, and severity was classified by Blantyre Coma Score [40]. The majority of patients were treated with an antimalarial drug (majority received quinine) within the 24 hours prior to admission. Parents or guardians of all children enrolled in the study were consented in writing in their own language by local native-speaking healthcare staff (nurse or doctor). A venous blood sample was drawn at admission and a 500 μl sample of whole blood was added directly to Tri-Reagent BD (Molecular Research Center, Cincinnati, OH, USA), mixed vigorously and stored at −80°C until processing.

### *In vitro P. falciparum* culture

The following *P. falciparum* lines were used in this study: P2G12, a gametocyte-producing clone from the reference strain 3D7 [41]; a transgenic line (termed 164/TdTom in the P2G12 background) expressing the tandem tomato fluorescent reporter under the control of the gametocyte-specific gene *PF10_0164* [42]; and the *P. falciparum* isolate CS2 [43]. Culture conditions were as described previously [44], maintaining parasites in O+ blood at 4% hematocrit in RPMI-1640 media supplemented with 10% human serum. Cultures were kept at 37°C in a chamber containing mixed gas (5% CO_2_, 5% O_2_, 90% N_2_).

### *In vitro* gametocyte formation and isolation

#### Production of sexually committed schizonts

For the generation of schizont samples for subsequent flow sorting we used the transgenic 164/TdTom line. Prior to induction of sexual commitment, asexual parasite cultures were synchronized for two cycles with 5% D-sorbitol [45]. To induce a maximal number of sexually committed schizonts, parasites were grown to a high parasitemia in the presence of partially spent (‘conditioned’) medium. Specifically, highly synchronous ring stage parasites (0 to 2 h post-invasion) were seeded into multiple T75 flasks 5 days prior to flow sorting, at a starting parasitemia of 0.1 to 0.25%. P2G12 wild-type and fluorescent 164/TdTom parasite lines were cultured alongside, in order to be able to properly gate the non-fluorescent population in preparation for flow sorting. To induce sexual commitment, half of the medium was changed daily and 17 h prior to sorting (at around 28 h post-invasion) parasites were stressed by doubling the medium volume [41,46]. For flow sorting late schizont stage parasites were separated from uninfected RBCs using a Percoll gradient. *P. falciparum* infected RBCs were washed and resuspended in RPMI medium without Phenol-red. Cells were subsequently stained for 30 minutes with 0.5 μM Vybrant DyeCycle Violet stain (Invitrogen, Eugene, OR, USA), which has fluorescence excitation and emission maxima of 369/437 nm, respectively, in complex with DNA.

### Flow sorting of schizont samples and cytospin analysis

A FACSAria II flow cytometer (BD Biosciences, San Jose, CA, USA) equipped with a combination of 407 nm, 488 nm, 561 nm, and 640 nm lasers was used for flow cytometry analysis and cell sorting. All experimental procedures with live cells were performed according to biosafety BL2+ level practice. To avoid the sorting of cell doublets or cell aggregates; single cells were sequentially gated based on FSC-H/FSC-W and SSC-H/SSC-W. Gating of fluorescent versus non-fluorescent schizonts was then done based on nuclear content using Vybrant Violet dye and TdTom fluorescence, with the wild-type parasite as a negative control.

For flow sorting, cells were collected in parallel from fluorescent and non-fluorescent schizonts of the stressed cells prepared from the 164/TdTom line. To confirm that only schizont stages were isolated, *P. falciparum* populations were subjected to Cytospin analysis after flow sorting. Specifically, Cytospin slide centrifugation was used to concentrate 100 μl of sorted parasite sample for Giemsa staining. Each sample was pipetted into a plastic chamber, placed in a cytospin slide centrifuge (Cytospin 2, Shandon Southern Instruments, Inc., Sewickley, PA, USA) and spun down for 5 minutes at a set speed of 100 rpm. Parasites were deposited in a 7 mm circular area on the slide, air dried and stained with Giemsa for 15 minutes. Cytospin smears were subsequently investigated under a light microscope (Axiostar plus, Zeiss Inc., Thornwood, NY, USA) and photomicrographs were taken.

Flow sorted cells were transferred directly into RNA lysis buffer (RNAeasy Micro Kit, Qiagen, Hildesheim, Germany) and subsequent RNA preparation was carried out according to the Manufacturer’s instructions. The eluted RNA was subjected to DNAse treatment using RQ1 RNAse-free DNAse (Promega, Madison, WI, USA), followed by another round of purification and elution into water. RNA quality was assessed by Bioanalyzer (Agilent 2100 Bioanalyser RNA 60000 Nano), and high quality RNA samples were labeled and hybridized to an oligonucleotide array (Affymetrix) custom-designed for the *P. falciparum* 3D7 genome, as published previously [32].

### Microarray expression assays

#### Sorted gametocyte microarray analysis

The raw CEL files were condensed into GCT expression files using RMA and the default parameter settings in ExpressionFileCreator in GenePattern [47]. Microarray data were then analyzed to define the subset of genes that are differentially expressed between the fluorescent and the non-fluorescent parasite populations. Expression fold change of each gene was calculated as ratio of mean of unlogged expression of each dataset. Any gene with two or greater fold change in the fluorescent population was annotated as sexually committed whereas any gene with 0.5 or less fold change was annotated as asexually committed. These data are shown in Additional file 4.

Please not that the CEL files have been deposited with Gene Expression Omnibus and are accessible under entry GSE64887.

### Co-expression with *ap2-g* (*PFL1085w*)

Distance, defined as Fisher transformation of Pearson correlation, between *PFL1085w* and each gene was calculated within each sample (*in vitro* time point, patient sample) individually, z-score normalized, and averaged across datasets following the same procedure as in network construction. Any gene with standardized distance less than −1.64 (the Z-value corresponding to a one-sided 0.05 significance level) was defined to have significant association with *PFL1085w*. Then, the per-cluster enrichment analysis procedure for the resulting gene set was performed as described above with FDR correction. These data are shown in Additional file 5.

### Temporal life cycle staging of field samples

The tight 52-hour asexual time course by Bozdech *et al.* [20] was used as the reference to estimate parasite stage (hours post-invasion) of patient samples. A cubic polynomial was fitted to the time course data for each gene after z-score normalizing each array. Each patient sample was also normalized separately and compared with the fitted curve. Parasite stage was defined as the time at which the mean squared difference between the fitted polynomial and the patient’s genome-wide expression was minimized.

### Quantitative reverse-transcriptase PCR for *in vivo* marker validation

#### *Primer design for novel* P. falciparum *marker genes*

Primers were designed using PrimerExpress software (Life technologies, Grand Island, NY, USA) and following recommended guidelines for quantitative reverse-transcriptase PCR (qRT-PCR) primer design for primers *PF14_0744* (cluster 44) and *PfAMA1*, and sentinel markers for variant group 1 (*PF14_0752*, *PF11_0512*, *PFL2565w* and *PFB0900c*) and group 2 (*PFE0060w* and *PFB0095c*). In addition all primers were checked for homology against *Plasmodium* or human homologous sequences using PlasmoDB and NCBI Blast in order to eliminate the chances of non-specific amplification (see also Additional file 6 for primer validation). Additional primers used in this study have been published previously [19,48].

### RNA extraction, DNAse digest and reverse transcription

RNA from *in vitro* cultures and patient samples was stored in TriReagent (Molecular Research Center) until use. For sample processing, RNA was extracted by an initial chloroform separation step. The RNA layer was then processed using the RNeasy mini kit (Qiagen) followed by DNAse digest (Ambion Life technologies, Grand Island, NY, USA). The quality of the RNA was determined on a 1% agarose, formaldehyde RNA denaturing gel and by Nanodrop. For first strand synthesis we used the SuperScript III First Strand Synthesis kit (Invitrogen). qRT-PCR assays were run on Applied Biosystems instrument using SYBR green (BioRad, Waltham, MA, USA)).

### qRT-PCR assay optimization

Amplification of the correct target sequence was confirmed by gel electrophoresis and melt curve analysis using SYBR Green (BioRad). Primer pair efficiencies were determined by calculating the slope of the crossing threshold (CT) values on 10-fold serial dilutions of mixed stage gDNA (Additional file 6).

### Gametocyte marker quantification

The expression levels of *PF14_0744*, *PF14_0748*, and *Pfs48/45* were compared against those of *Pfs25*, *PfAMA1* and *Ubiquitin conjugating enzyme* (*UCA*) [19]. First an overall Kruskal-Wallis test was performed to check if at least two of the genes were differentially expressed. Then pair-wise permutation *t*-tests were performed to compare the transcript levels of *PF14_0744* and *PF14_0748* against those of *Pfs48/45*, *Pfs25*, and *PfAMA1* (10,000 permutation per test, Bonferroni correction)*.*

## Results

### Reconstruction of a functional *P. falciparum* gene network identifies groups of highly connected parasite-stage-specific clusters

**Figure 1:**
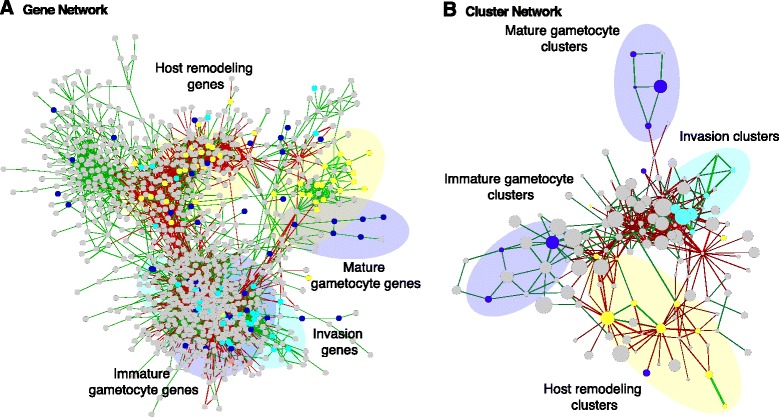
**Reconstruction of a functional** ***P. falciparum*** **transcriptional network. (A)** Gene network. Genes (nodes) are connected by edges indicating functional interactions, as defined by normalized, meta-analyzed co-expression across nine *in vivo* and *in vitro* datasets. For visualization, the highest 0.1st percentile of normalized co-expressions (edges) is shown, and singleton genes that would result from this filter are omitted. Genes with gametocyte, host cell remodeling or host cell invasion annotations (Additional file 1) are marked in blue, yellow and turquoise, respectively. Note that immature and mature gametocytes form separate sub-networks. **(B)** Network of gene clusters. Clusters (functional modules) of tightly linked genes were defined in the gene network (see Methods) and used as a convenient summarization for analysis throughout this work. Shown is an overview of how these clusters relate to each other, as defined by averaging all edges between genes spanning each cluster pair, using the same color-coding as in (A). Circle sizes indicate the relative number of genes per cluster.

We constructed a genome-wide network of co-expressed genes in *P. falciparum* that incorporates information from three *in vivo* datasets [25,26,29] and six *in vitro* time courses [20,30-32], together totaling over 573 expression conditions (Figure 1A). Briefly, all pairwise correlations among genes within each dataset were computed, normalized to z-scores, and the resulting per-dataset co-expression values were meta-analyzed by averaging across datasets to provide a single global network [34,35] (see Methods). Previous *P. falciparum* gene networks are based on the analysis of co-transcription during the asexual parasite cycle and under controlled *in vitro* conditions only [23,24]. Our aim was to generate an expanded functional network by integrating asexual and gametocyte *in vitro* time courses, as well as transcriptional profiles from more than 100 clinical parasite isolates collected from two cohorts of uncomplicated malaria in Senegal and one cohort of cerebral malaria in Malawi [25,26,29]. Our approach allowed us to retain information from asexual stages and gametocytes *in vitro*, while also adding information on co-transcribed genes and the activity of functional modules during human infection. Comparison with previously published networks demonstrated overlap in many conserved processes, while our network additionally includes novel information on gametocyte development and on host-specific processes (Additional file 7).

We identified 284 modules of co-expressed genes within this network, that is, clusters that represent putative pathway components, complexes, or other functionally cohesive sets of factors co-regulated during at least one stage of the IDC (Figure 1B). Cluster enrichment was assessed by Kruskal-Wallis tests or, for continuously valued clinical phenotypes, by Spearman correlation. Significance (q-value ≤0.05) was evaluated after FDR correction for multiple hypothesis testing (see Methods). Any cluster containing at least five genes (see Methods; Additional file 1) was given a numerical identifier and annotated with enrichment for the following attributes (Additional files 1 and 2): i) GO terms, as provided by the Bioconductor package org.Pf.plasmo.db in R; ii) gametocyte-specific expression, using our recent [29] re-analysis of gametocyte temporal profiles [30]; iii) host cell remodeling, based on presence of a protein export motif [37]; iv) host cell invasion, based on gene annotations in PlasmoDB; and others as discussed below. Most of the clusters in this network contain a relatively small number of genes, with a median and maximum cluster size of 13 and 71, respectively. As with the overall gene network comparison above, we recovered clusters similar to almost all of those extracted from previous *in vitro* networks, while also identifying new functional modules (Additional file 7).

The combination of a large number of diverse datasets and a conservative process to define modules in our network allowed us to assign a putative function to many of the clusters with high confidence. We identified a total of 16 clusters that are most significantly enriched in either young or immature gametocyte annotations and 9 that are most significantly enriched in mature gametocyte traits (q-value ≤0.05 in both cases). Several of these clusters include previously characterized gametocyte-specific genes (Additional files 1 and 8). For example, cluster 44 includes the young gametocyte markers *PF14_0744* and *PF14_0748* [49,50], while clusters 36, 49 and 67 contain genes encoding proteins with known functions during early stages of mosquito infection. Many clusters are also associated with distinct pathway annotations. Cluster 30, for example, is enriched both in mature gametocyte genes and in genes controlling microtubule-dependent functions, suggesting that this set of genes plays a role in male gametocyte exflagellation. While 5 of the 13 genes in this cluster define pathway enrichment, 7 factors are yet missing functional annotation and it will be interesting to consolidate their likely involvement in male gametocyte maturation in future studies. Generally, our network allowed us to assign putative roles to many genes of unknown function in *Plasmodium*. Additional file 1 lists all clusters, their corresponding gene content, and a per-cluster enrichment score (significance at a q-value ≤0.05) for specific attributes; the corresponding GO term enrichment scores are given in Additional file 2.

Given the degree to which *in vivo* data were newly incorporated into our network, clusters with a putative role in host cell interactions were of particular interest in this analysis. We found 18 clusters to be significantly enriched (q-value ≤0.05) in proteins with a predicted export motif [2,3]. Excluding the three most polymorphic gene families, *var*, *rif* and *stevor* (which are insufficiently represented in microarray platforms to allow for meta-analyses), these clusters combine the vast majority of the previously predicted ‘exportome’ in *P. falciparum* [37]. Additionally, we identified a total of 7 clusters enriched (q-value ≤0.05) in factors associated with host cell invasion. Cluster 277 includes the invasion ligands of the erythrocyte binding antigen (EBA) and reticulocyte binding-like protein homolog (RH) families, and while cluster 266 contains many rhoptry-associated proteins, merozoite surface proteins as well as myosin A and its interacting factor MTIP are prominent components of cluster 275. Some gene sets are particularly highly specialized, such as cluster 38 (enriched in Maurer’s clefts proteins) and cluster 19 (enriched in components of the *Plasmodium* translocon for exported proteins, PTEX).

Altogether, 60 of our 284 clusters (21.1%) retained significant enrichment (q-value ≤0.05) for one or more host-associated features or for gametocyte development.

### Comparative cluster analysis defines asexual parasite sequestration dynamics

**Figure 2:**
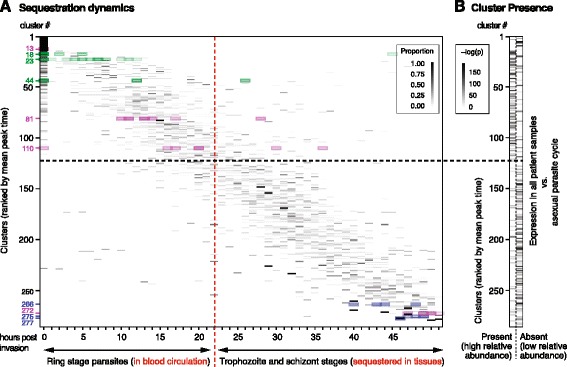
**Asexual sequestration dynamics and differential gene expression during infection. (A)** Distribution of asexual peak time across transcriptional clusters. Each row represents one cluster, and each column shows a 2-hour interval during the asexual parasite life cycle [20]. Shading intensity indicates the distribution of individual gene peak times within each cluster. Clusters are ranked vertically by increasing overall peak time during the asexual parasite cycle from top to bottom. Clusters of particular interest are highlighted and color-coded: export clusters, green; invasion clusters, blue; gametocyte-specific clusters, purple. **(B)** Asexual peak time versus parasite circulation properties during infection. For each cluster, mean transcript abundance in the patient samples versus asexual *in vitro* time courses was calculated by *t*-test to determine differential expression levels (see Methods; Additional file 1). Clusters are ranked as in (A), demonstrating that the vast majority of clusters peaking at ≤22 hours post-invasion are expressed in patient samples (representing clusters of circulating parasites) while most of those peaking later are down-regulated (representing clusters of sequestering parasites). The intersection of the two dashed lines represents the inflection point between ‘circulating’ and ‘sequestering’ clusters.

We next took advantage of our network’s combination of *in vitro* and *in vivo* parasite biology to study dynamics and potential molecular mechanisms of asexual parasite sequestration. A hallmark of *P. falciparum* is its capability to sequester in the microvasculature of deep tissues during asexual development in human RBCs [4]. While ring-infected RBCs are present in circulation, later asexual parasite stages (termed trophozoites and schizonts) sequester and are therefore absent from circulation. As patient blood samples only contain circulating parasites (except after antimalarial treatments and in splenectomized patients), we hypothesized that transcripts of genes with peak expression in a sequestered stage should be less prominent (or absent) in the patient samples when compared with *in vitro* data. To test this hypothesis and thereby define the sequestration dynamics of asexual parasites, we determined peak expression for each cluster based on the asexual *in vitro* time courses used in this study. This was defined as the mean of the individual peak times of all *P. falciparum* genes within the cluster (Figure 2A). Genes showing maximal activity both at the end of one cycle and immediately after re-invasion were assigned with a peak time of 0 to 2 hours post-invasion, explaining the accumulation of genes within this time frame. In parallel, we also measured differential gene expression between all the *in vitro* and *in vivo* datasets to determine whether clusters are associated with genes transcribed during a sequestered (absent) or circulating (present) parasite stage (Figure 2B).

Clusters with a mean peak time later than 22 hours post-invasion showed a dramatic decrease in transcript abundance in the *in vivo* datasets (Additional file 1), confirming previous transcriptional evidence that circulating asexual parasites represent the first approximately 20 hours of development only [51]. It is noteworthy, however, that this is the first time that these dynamics have been thoroughly assessed *in vivo* (that is, during infection). Because of their direct or indirect involvement in host cell remodeling and tissue sequestration, we expected exported proteins to be expressed early during the asexual cycle. Indeed, all but two clusters enriched in these factors had a mean peak time of ≤22 hours. In contrast, we found that transcriptional activity of clusters enriched in invasion factors peaks later during the asexual parasite cycle, reflecting the need of trophozoite and schizont stages to prepare for subsequent re-invasion. Examples for this distinct distribution of functional gene set activity are given in Figure 2A: whereas the abovementioned invasion clusters 266, 275 and 277 (marked in blue) show activity late during the IDC, genes found within the export clusters 18, 23 and 44 (marked in green) are transcribed early.

**Figure 3:**
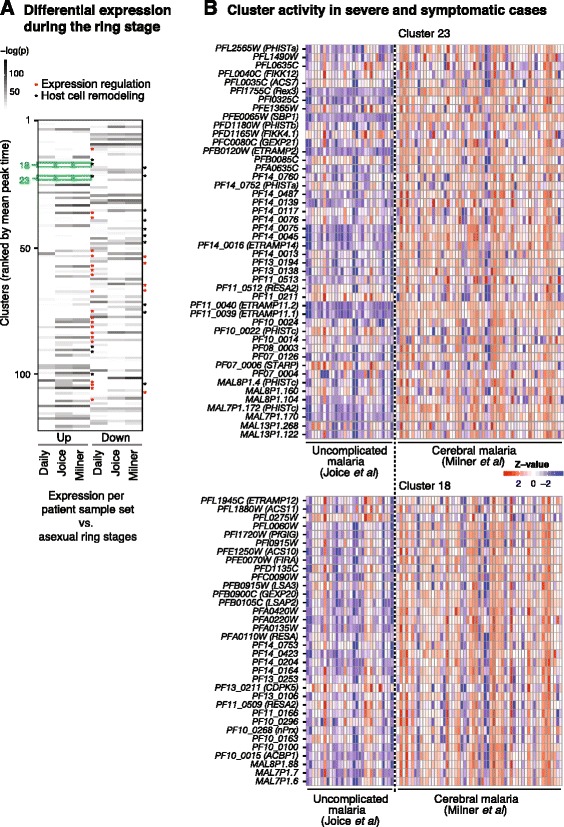
**Individual gene expression values from representative clusters associated with parasite phenotypes. (A)** Differential expression of ring stage clusters. Based on the mean transcript abundance as calculated in Figure 2, differential expression of ring stage clusters (mean peak time ≤22 hours) was determined by combined t-statistic (see Methods) of *in vitro* parasite time points between 0 and 22 hours post-invasion versus each *in vivo* dataset. Most clusters that are up-regulated in patient isolates are enriched in genes involved in expression regulation (transcription, translation, protein degradation; red asterisks), while some clusters enriched in exported proteins are up-regulated in patient samples and others are down-regulated (black asterisks). Clusters are ranked as in Figure 2. **(B)** Differential gene expression between patient cohorts. Each row corresponds to one gene, and each column represents a patient sample. Clusters 18 and 23 are up-regulated in isolates from patients with cerebral malaria compared with those from patients with uncomplicated malaria (aggregate t-statistic across genes in cluster). These clusters are highly enriched in secreted parasite antigens, including RESA, RESA2, LSA3, LSAP, ACS11, and GEXP20 as well as several ETRAMP and PHIST proteins.

We further investigated which asexual clusters are differentially expressed between infection and *in vitro* culture by comparing the *in vitro* transcriptomes with each of the three field datasets separately (Figure 3A). Previous studies have demonstrated altered transcriptional profiles during infection, representing responses to starvation and environmental stress [25]. Also, genes encoding exported parasite antigens, including the *var* genes, show reduced activity during *in vitro* culture [27,28]. To identify such differential expression, we compared the per-cluster transcriptional activity of field samples with corresponding *in vitro* gene expression (see Methods). We performed the analysis separately for patient samples from Senegal [25,29] and Malawi [26]. As the *in vivo* datasets include information about circulating parasites only, this comparison was restricted to clusters with a peak time ≤22 hours post-invasion [26]. A total of 24 clusters showed significant enrichment (q-value ≤0.05) in genes expressed in all the three field cohorts compared with *in vitro* ring stage parasites (Figure 2B), and a small subset of those was also differentially expressed between these cohorts (Figure 3A).

Interestingly, the majority of clusters with differential expression during infection were significantly enriched (q-value ≤0.05) in genes involved in transcriptional and translational processes (Additional files 1, 2 and 8). Moreover, five clusters were significantly enriched (q-value ≤0.05) in exported proteins (clusters 16, 18, 23, 38 and 101), and they contain many of the factors essential for Maurer’s cleft structure, knob formation, PfEMP1-mediated adherence and maintenance of host cell rigidity [52]. Genes in clusters 18 and 23, for instance, showed higher expression in patients with cerebral malaria than in cases of uncomplicated malaria. These clusters harbor various factors with a putative function at the host-parasite interface, such as FIKK kinases and various members of the exported PHIST and ETRAMP proteins (Figure 3B). Noteworthy, several proteins in these two differentially expressed clusters are required for parasite virulence phenotypes such as endothelial adherence (for example, MAL7P1.172, PFE0065w) or cellular rigidity (RESA) [52,53], supporting their potential role in disease severity (Figure 3).

### Patterns of expression variation *in vitro* and *in vivo*

**Figure 4:**
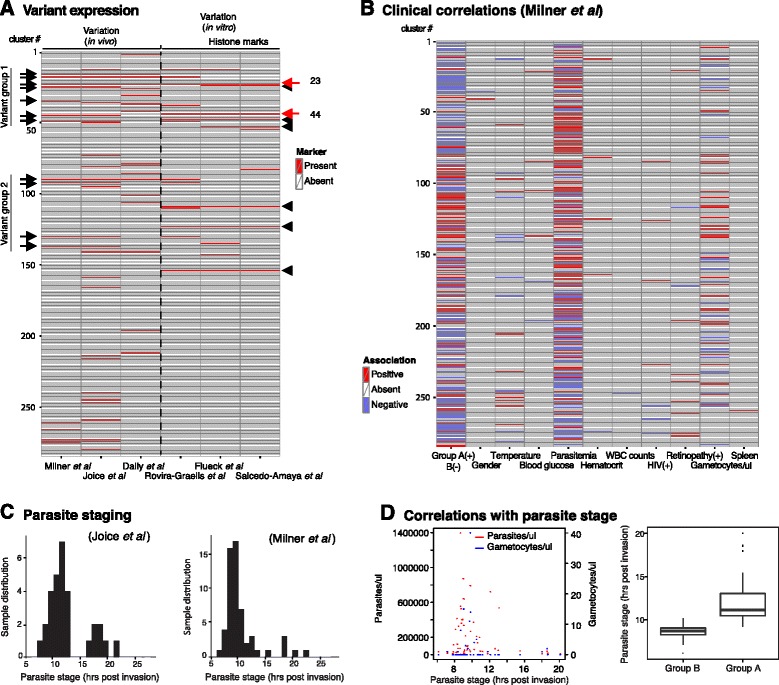
**Clusters with variant expression and association with clinical phenotypes. (A)** Measures of variant expression *in vivo* and *in vitro*. Shown is a heat map with clusters enriched in variantly expressed genes based on both field and *in vitro* arrays (left), or based on H3K9m^3^ histone modification (right) (see Methods; Additional file 1). Note that only clusters 23 and 44 share all the three phenotypes, as marked with red arrows. Clusters with variant expression pattern across patient samples in the Malawi cohort are marked with black arrows. Black arrowheads: clusters with enrichment in genes with H3K9m^3^ marks. **(B)** Asexual parasite stage in field samples. Distribution of parasite stages in the Senegal (left) and Malawi (right) cohorts shows that the majority of parasites in both cohorts are at 10 h post invasion. **(C)** Clinical phenotypes and correlations with clusters. Shown are those clinical parameters from the Malawi cohort with at least one significant cluster association (q-value ≤0.05 by aggregate Kruskal-Wallis statistic or Spearman correlation; see Methods, Additional file 9). **(D)** Correlations with parasite stage. Parasitemia and gametocytemia are shown for each sample (left panel). Stage distribution of genes in the two transcriptional groups (A and B) as defined by Milner *et al*. [26] is presented in the left panel. WBC, white blood cell.

Several studies demonstrated that phenotypic variation in *P. falciparum* virulence (for example, cytoadherence, host cell invasion) and transmission (for example, gametocyte formation, mosquito infection) pathways can be detected and quantified using transcriptional approaches (for example, [8,13,29]). We used our annotated transcriptional network to investigate this variation separately *in vitro* and *in vivo*. First, we measured per-cluster enrichment of genes associated with H3K9m^3^ histone marks that are diagnostic for epigenetic gene regulation [11,38]. We identified eight clusters significantly enriched (q-value ≤0.05) in H3K9m^3^-demarcated genes, as defined by Flueck *et al*. [11] and Salcedo-Amaya *et al*. [38]. All but one of these clusters were also significantly enriched (q-value ≤0.05) in virulence genes encoding exported proteins, including many paralogs of the epigenetically regulated *Pfmc-2TM*, *fikk* kinase, acyl-CoA synthase, *phista* and *phistb* gene families (Figure 4A). Interestingly, the young gametocyte cluster 44 is also significantly enriched (q-value ≤0.05) in genes associated with H3K9m^3^, supporting the recent finding that gametocyte formation is epigenetically regulated [17,18]. Second, we measured per-cluster enrichment of genes that are variantly expressed across *in vitro* cultured clones [13] and identified 14 clusters (Figure 4A). Amongst those, six clusters also showed significant enrichment (q-value ≤0.05) of genes with H3K9m^3^ marks [11,38], demonstrating that epigenetic variability can be measured under controlled *in vitro* conditions.

We next determined transcriptional variation across patient isolates. Genes with variant expression during infection were defined as those with a variance greater than the 20th percentile of all variances after excluding constitutively expressed and low expression genes in the *in vivo* datasets (see Methods). We identified 16 clusters significantly enriched (q-value ≤0.05) in variantly expressed genes based on the cerebral malaria cohort samples from Malawi [26], and the majority of those also showed enrichment in the cohorts from Senegal [25,29] (Figure 4A). Surprisingly, only six of these clusters overlapped with enrichment in the ‘variantome’ [13], and only two were also enriched in H3K9m^3^-bound genes. Instead, several variant clusters were significantly enriched (q-value ≤0.05) in genes coding for exported proteins and two represented mature gametocyte clusters. We observed that the export gene clusters are activated at either of two distinct time frames during the asexual cycle in very young ring stages, at 4 to 10 hours post-invasion, and in later ring stages at 17 to 20 hours post-invasion (Figure 4A). The existence of these two ‘variant groups’ was confirmed by qRT-PCR on samples of a separate malaria cohort from Blantyre, Malawi (Figure S2A in Additional file 10). Considering the highly stage-specific expression pattern of genes encoding exported proteins [2,20], we wanted to determine whether the apparent variant expression pattern across patients’ co-expression might be driven by differences in parasite life cycle stages between samples. Supporting this hypothesis, our qRT-PCR approach revealed that genes within a certain variant group are co-transcribed across patients, but anti-correlated between the groups. We have recently developed a method to define parasite stage composition in patient samples [29], and a similar method has been described to determine the mean parasite stage in a sample [51]. Using the former approach, we estimated parasite stage for all samples in the patient cohorts from Senegal [25,29] and Malawi [26] (Figure 4B). Sorting patient samples by estimated parasite stage confirmed that the variant expression pattern observed was indeed driven by differences in parasite stage: clusters in variant group 1 were up-regulated in patient samples with early parasite stage and those in variant group 2 were up-regulated in samples with later parasite stage (Figure S2B in Additional file 10). In summary, variant expression of epigenetically regulated, H3K9m^3^-demarcated genes is detectable under controlled *in vitro* conditions. In patient samples, such patterns may be masked by differences in parasite stage across samples, while variant expression as a result of variable gametocyte levels in a sample can be detected *in vitro* and across patients.

### Expression variation and clinical phenotypes

The identification of regulatory programs activated in response to distinct *P. falciparum* physiological states during human infection suggested that a parasite population could quickly adapt to the host environment [25]. These changes may in turn affect the clinical outcome. For example, differential expression of particular PfEMP1 variants results in tissue-specific parasite sequestration and is linked to distinct pathology such as cerebral or pregnancy-associated malaria (for example, [4]). Previous characterization of cerebral malaria patient samples from Malawi identified two large transcriptional gene sets (previously termed groups A and B) that differentiate high and low parasitemia infections, respectively [26]. We wanted to determine whether transcriptional clusters as defined in our study showed significant association (q-value ≤0.05) with parasitemia or other clinical phenotypes documented in this cerebral malaria cohort.

After controlling for stage bias (see Methods), we tested each cluster for enrichment in genes associated with a variety of clinical parameters, including parasitemia, gametocytemia, fever, white blood cell count and gender (Figure 4C; Additional file 9). As expected, the activity of most gametocyte clusters showed significant (q-value ≤0.05) positive correlation with gametocyte counts by microscopy. By contrast, we generally found a significant negative correlation (q-value ≤0.05) between gametocyte cluster activity and parasitemia, probably reflecting natural infection dynamics where gametocyte development is triggered late during disease progression [19]. We also noted that retinopathy, which serves as a proxy for cerebral malaria, was positively correlated (q-value ≤0.05) with gene expression in clusters 275 and 277. These two main invasion clusters contain the majority of the structural and functional determinants of parasite invasion into RBCs. It is likely that this reflects increased replication rate as these clusters are also positively correlated with parasitemia. Surprisingly, transcriptional groups A and B [26] showed a bias towards late (patient group A) and early (patient group B) ring stages, respectively (Figure 4D). This strongly suggests that the previously defined distinct transcriptional profiles of these patient samples are at least partially driven by differences in parasite staging.

### The transcriptional profile of sexual commitment and young gametocytes

We have recently shown that gametocyte development takes place in the hematopoietic system of human bone marrow [19]. However, due to the absence of specific transcriptional markers, it remained unclear whether this is also the site of gametocyte formation or whether very young gametocytes (that is, deformable gametocyte rings) are in circulation before homing to the bone marrow. In analogy to the approach used to define asexual sequestration dynamics (Figure 2), we investigated the differential expression pattern of gametocyte clusters across peripheral blood samples to define gametocyte sequestration dynamics during development.

**Figure 5:**
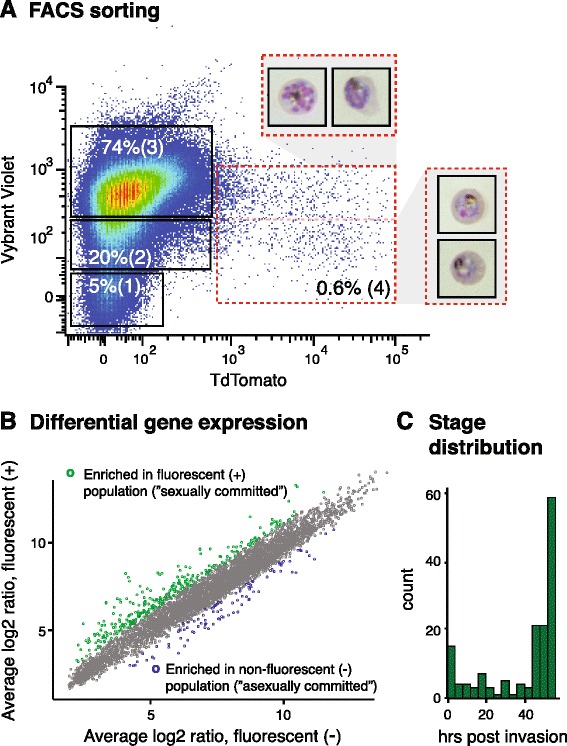
**Transcription during early gametocyte development. (A)** Flow sorting of putative sexually and asexually committed parasites. Synchronized and stressed schizonts of the transgenic 164/TdTom line were collected and purified at around 45 hours post-invasion, and separated by flow cytometry. Infected RBCs are gated based on nuclear content using the nuclear dye Vybrant Violet, and sexually committed parasites including gametocytes are gated based on the TdTomato signal. Shown is one of two biological replicates containing 5% uninfected RBCs (1), 20% non-fluorescent parasites with a single nucleus (2, trophozoites), 74% non-fluorescent parasites with multiple nuclei (3, schizonts) and 0.6% fluorescent parasites with single and multiple nuclei (4; gametocytes, putative sexually committed schizonts). From populations 3 and 4 a total of 5 × 10^5^ to 10^6^ cells were collected for microarray analysis, including an aliquot for cytospin to confirm stage composition (see inserted picture of representative schizonts and gametocytes)*.* **(B)** Affymetrix microarray analysis of putative sexually and asexually committed parasites. The scatterplot is based on data from two biological replicates of stressed schizont populations that were subsequently enriched and sorted. Genes with a mean differential expression level of ≥2-fold are marked in green for those up-regulated in the fluorescent population (‘sexually committed’; 305 genes) and in blue for those up-regulated in the non-fluorescent population (‘asexually committed’; 98 genes). **(C)** Staging of sexually committed cells. The histogram shows the distribution of transcriptional peak times for genes up-regulated in the fluorescent population (‘sexually committed’) in Figure 5B.

The published gametocyte time course we used for network generation [30] omits the first 24 hours of sexual development as well as the commitment stage in the previous asexual cycle. We hence generated a new transcriptional profile of these earliest steps of gametocyte development. Specifically, we used a transgenic parasite line expressing a red fluorescent protein reporter under a gametocyte-specific promoter for isolation of sexually committed schizonts by fluorescence activated cell sorting (FACS; Figure 5A). We have previously demonstrated that the promoter of the highly expressed gametocyte-specific ETRAMP 10.3 (accession number PF10_0164) is able to drive reporter expression (Tandem tomato, TdTomato) in a subset of schizonts and across gametocyte development, including mature stage V gametocytes [41,42]. To produce a culture enriched in sexually committed schizonts, we stressed a highly synchronous trophozoite population of the transgenic parasite line 164/TdTom [42] by addition of conditioned medium as described previously [41,46]. We subsequently isolated 5 × 10^5^ to 10^6^ mature schizonts from both the fluorescent and non-fluorescent population by FACS and prepared for microarray analysis. Notably, the fluorescent population also contained a fraction of developing gametocytes, as represented by low DNA content and characteristic morphology of parasites as determined after preparation by cytospin (Figure 5A).

Differential gene expression analysis of two biological replicates revealed induction of a subset of genes in each population (Figure 5B; Additional file 4). Specifically, we identified 308 genes that were at least two-fold induced in the fluorescent, sexually committed population compared with the non-fluorescent population. Within this set of genes we detected all of the known young gametocyte markers, including *Pfs16* (6.7-fold up-regulated) [54], *Pfg27* (6.2-fold up-regulated) [55], as well as the two markers from cluster 44, *PF14_0744* (9-fold up-regulated) and *PF14_0748* (6.6-fold up-regulated) [49,50]. Moreover, the gene encoding transcription factor AP2-G (*PFL1085w*), a recently identified master regulator controlling the onset of sexual differentiation [15,16], was up-regulated four-fold, corroborating enrichment of sexually committed schizonts and very young gametocytes in the fluorescent population. The majority of genes up-regulated in the sexually committed population showed peak transcription during the schizont stage (Figure 5C), confirming that we were able to track steps during sexual commitment.

To independently identify markers of sexual commitment and early gametocyte development, we also searched for genes with a similar transcriptional variation across all samples as the commitment marker *ap2-g* (that is, *PFL1085w* co-expression; Additional file 5). This gene is under epigenetic control, and it has recently been demonstrated that genetic knock-down of one of its regulators, *P. falciparum* heterochromatin protein 1 (PfHP1), de-represses *ap2-g* transcription and greatly increases gametocyte production on a population level [18]. Transcriptional profiling of these PfHP1 knock-down parasites identified a set of differentially expressed genes, including known and putative young gametocyte markers [18] (Additional file 3).

### Young gametocyte clusters and the dynamics of gametocyte sequestration

We used our newly generated profile of sexual commitment together with the analysis of *PFL1085w* co-expression and the PfHP1 knock-down experiments as three additional annotations to further define the transcriptional network and identify clusters enriched during sexual commitment and early gametocytogenesis (see Methods). Noteworthy, residual gametocyte production in the published asexual transcriptome allowed us to assign peak times to those gametocyte clusters with peak expression within the first 48 hours of the developmental cycle. We identified 19 clusters significantly enriched (q-value ≤0.05) in genes of at least one of the three annotations (Additional file 1). Six of these clusters (clusters 224, 249, 255, 256, 266 and 272) had mean peak times between 41 and 51 hours post-invasion, suggesting that these are putative committed schizont clusters. Interestingly, cluster 272 contains many molecules involved in intracellular signaling, including protein kinase A (PKA) and adenylate cyclase, two components of a conserved cAMP-dependent signaling cascade that has previously been implicated in regulation of gametocyte formation [56,57]. These factors, together with other molecules in cluster 272, may integrate and translate external stimuli into a signal for sexual differentiation. Clearly, this hypothesis will require experimental proof. However, we consider the concerted up-regulation of genes in cluster 272 as further initial evidence for the successful profiling of the earliest steps of sexual development. We also identified four clusters (clusters 13, 20, 81 and 110) with mean peak times between 0 and 20 hours post-invasion, suggesting that these clusters are active in very young gametocytes. Indeed, these clusters contain many known markers of early gametocyte development: *Pfs16* and *GEXP05* [58] in cluster 13, *GEXP02* [59] in cluster 81 as well as *GEXP04* [58] and *puf1* [60] in cluster 110. In addition, the immature gametocyte cluster 44, containing the two known young gametocyte markers *PF14_0744* and *PF14_0748*, showed the greatest enrichment in genes differentially expressed in these commitment arrays. Based on their enrichment and the early peak times, we annotated these five clusters as putative ‘gametocyte ring’ clusters.

**Figure 6:**
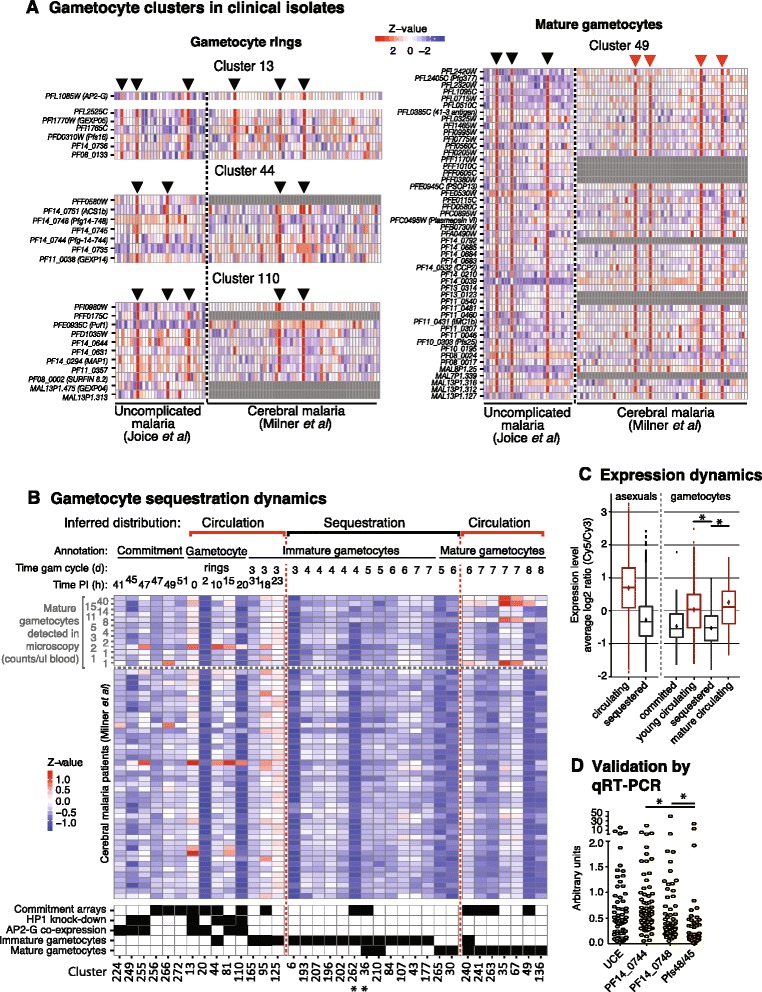
**Dynamics of gametocyte sequestration. (A)** Gametocyte ring and mature gametocyte clusters. Left panel: gametocyte ring clusters 13, 44 and 110 show expression in the same subset of isolates from two patient cohorts. The commitment-specific transcription factor *ap2-g* (*PFL1085w*) is active in the same individuals, demonstrating presence of circulating gametocyte rings in this subset of patients. Right panel: genes from the mature gametocyte cluster 49, including the gold standard gametocyte marker *Pfs25*, are co-expressed in the same seven patients. Red arrowheads mark isolates with slide positivity for gametocytes (data available for Milner *et al*. [26] only). **(B)** Relative expression of gametocyte clusters *in vivo*. Expression dynamics in samples from cerebral malaria patients in Malawi [26]. Gametocyte clusters were separated into four categories based on the minimum q-value resulting from gene enrichment for: i) committed schizont, ii) gametocyte ring, iii) immature gametocyte (young and developing gametocytes), and iv) mature gametocyte markers (Additional file 1). Two clusters (36 and 262, marked with crosses) were manually reassigned due to significance in multiple categories and greater marker expression in a non-minimum category. Within these categories, clusters are horizontally sorted by peak time, as indicated above the heatmap: asexual peak time was used to map clusters of committed schizonts and gametocyte rings; for immature and mature gametocyte clusters the NF54 gametocyte time course by Young *et al*. [30] was used as reference. Transcriptional analysis of clusters indicated expression of four gametocyte ring clusters, two immature gametocyte clusters, and seven mature gametocyte clusters in a subset of patient blood samples. Predicted circulation and sequestration properties during gametocyte development are indicated above the cluster peak times. Slide positivity for each sample is indicated on the right. Numbers represent number of gametocytes per microliter based on smears. The heat map at the bottom highlights clusters significantly enriched (q-value ≤0.05) in putative gametocyte-specific genes, identified by four independent approaches, in black. **(C)** Expression dynamics of circulating and sequestering parasite clusters. To compare global expression levels of circulating and sequestering parasite populations, we analyzed mean transcript expression across clusters. For asexual parasites, mean transcript expression across circulating (peak time <22 hours post-invasion) versus sequestering (peak time >22 hours post-invasion) clusters was compared in samples from Milner *et al.* [26] and Joice *et al.* [19]. Mean transcript expression from clusters representing circulating asexual parasites was significantly higher than from sequestering ones (*P* < 10^−15^). For gametocytes mean transcript expression was determined from the subset of patient samples with detectable transcript levels. These are the six samples marked with black arrowheads in **(A)** (left) for clusters representing young circulating parasites (as defined in **(B)**), and the seven samples marked with black or red arrowheads in **(A)** (right) for clusters representing mature circulating gametocytes (as defined in **(B)**). For mean expression of clusters representing commitment and sequestering (immature) gametocytes the combined 13 samples from above were analyzed. Transcripts from both circulating young and mature gametocytes are detectable at significantly higher levels than the genes from immature gametocytes (*P* < 10^−10^), but they are not significantly different from each other (*P* = 0.3939). **(D)** Gametocyte marker quantification in blood samples by qRT-PCR showing 78 samples from a cerebral malaria cohort in Blantyre, Malawi. Sentinel markers for gametocyte ring cluster 44 (*PF14_0744*, *PF14_0748*) and immature gametocyte cluster 36 (*Pfs48/45*) and the constitutive marker *Ubiquitin conjugating enzyme* (*UCE*) [19] are shown. Overall comparisons of all groups showed a significant difference in at least two of these genes (*P* < 10^−15^, Kruskal-Wallis) and post-test pair-wise comparisons showed significant differences (*P* < 0.0001, Kruskal-Wallis) when comparing the transcript levels of *PF14_0744* and *PF14_0748* against that of *Pfs48/45.* Data are normalized by RNA input and values are shown as arbitrary units.

We analyzed *in vivo* gene expression across all 35 gametocyte clusters identified in this study (that is, from commitment to mature gametocytes) to define the sequestration dynamics during gametocyte development (Figure 6A,B). Expression analysis of the Malawi patient cohort samples [26] indicated that seven out of nine mature gametocyte clusters showed measurable expression within a subset of patients with mature gametocytes as detected by microscopy (Figure 6B). By contrast, only 2 out of the 15 immature gametocyte clusters showed measurable expression in any of the patients, supporting the idea that immature gametocytes are not present in the blood circulation. The two immature gametocyte clusters (clusters 95 and 125) with evidence for *in vivo* expression had among the earliest mean peak times within the set of immature gametocyte clusters (based on the NF54 gametocyte time course [30]). Moreover, four out of five putative gametocyte ring clusters showed expression in a specific subset of patient isolates (Figure 6A,B). The early gametocyte clusters detected in circulating parasites (clusters 13, 44, 81, 95 and 110) had a mean peak time of <20 hours post-invasion, suggesting similar gametocyte sequestration dynamics as those measured for asexual parasites (Figure 2). As expected, expression of the *ap2-g* sexual commitment marker, *PFL1085w*, showed the same pattern across patients as the early gametocyte clusters (Figure 6A). *In vivo* expression analysis demonstrated that transcripts of young and mature gametocyte clusters are detectable at a similar frequency in the circulating blood of malaria patients: six patient samples showed expression of young gametocyte (ring) clusters and seven patient samples showed expression of mature gametocyte clusters (Figure 6A). We also observed similar mean transcript levels across all genes for clusters representing young and mature gametocytes while levels differ significantly from those clusters representing (sequestering) immature gametocytes (Figure 6C).

Finally, we confirmed the presence of gametocyte ring markers in circulation using independent qRT-PCR experiments. Importantly, the higher sensitivity of this approach allowed us to resolve gametocyte-specific transcription in the large number of samples with gametocyte numbers below microarray detection limits. We quantified transcript levels of the known gametocyte markers in cluster 44, *PF14_0744* and *PF14_0748*, in a subset of blood samples from a separate cerebral malaria cohort in Malawi (Figure 6D). Indeed we detected these two markers, while the immature gametocyte marker *Pfs48/45* could only be detected in a small subset of samples. Altogether, these data provide the first direct evidence for the quantitative presence of very young gametocytes (that is, gametocyte rings) in circulation, thus supporting the hypothesis that commitment and the first steps of *in vivo* gametocyte development can occur outside of the bone marrow hematopoietic system*.*

## Discussion and conclusions

Human malaria represents one of the major public health issues worldwide. Current efforts to control or eliminate malaria are hampered by the lack of an effective vaccine and the rapid spread of drug resistance. Reasons for the unique capability of the parasite to rapidly respond to changing environments include transcriptional and genetic versatility as well as a phenomenal efficiency in infecting the *Anopheline* mosquito vector. We generated the most comprehensive transcriptional network of the *P. falciparum* RBC parasite stage to date by including more than 100 patient samples, a published gametocyte *in vitro* time course and the first set of sexually committed schizont samples. This allowed us not only to identify potential functional modules (or clusters) of co-expressed genes, but also to investigate differential expression between *in vitro* and *in vivo* conditions and to map the sequestration dynamics of the parasite during human infection. Individual genes (nodes) within our network were annotated with information ranging from GO [36] terms to clinical correlates, and these per-gene annotations were aggregated to detect significant enrichments per cluster. Among these functional gene sets, we defined a total of 35 transcriptional clusters that define gametocyte development from sexual commitment to mature stage V gametocytes.

Our integrated network defines potential functional relationships based on co-expression of *Plasmodium* genes, and thereby provides a rational basis for hypothesis-driven studies on parasite biology *in vitro* and during infection. Although we used a straightforward agglomerative clustering approach to simplify this genome-wide network into functional modules, this was for convenience only and could be refined or replaced to define more precisely delimited pathways for sequestration or variant gene regulation in the *in vivo P. falciparum* transcriptome. Likewise, these integrated data could be extended with, for example, physical protein-protein interactions [61,62] or information on evolutionary conservation [27] as the integration methods used are amenable to heterogeneous data [35,63].

To define asexual sequestration dynamics, we measured differential gene expression in the patient isolates and mapped the profiles along a high resolution *in vitro* time course previously published by Bozdech *et al.* [20]. The estimated circulation period of 22 hours fits well with the known dynamics of host cell rigidity and cytoadherence of infected RBCs. It is well established that ring-infected RBCs retain deformability properties of uninfected RBCs while the presence of more mature parasites significantly increases the rigidity of the host cell [64,65]. This switch in cellular rigidity coincides with induction of adhesive properties due to PfEMP1 surface display at around 20 hours post-invasion [66,67]. Transcriptional activity of the PfEMP1-encoding *var* genes is rapidly down-regulated once clinical isolates are cultured *ex vivo* [28], and such transcriptional and physiological changes of the parasite during culture adaptation are of concern when conducting *in vitro* studies.

Although the highly diverse *var* genes encoding PfEMP1 could not be captured in this analysis, we identified a large number of clusters that showed differential expression patterns in *in vitro* cultures compared with human infection. Many of these clusters were enriched in genes involved in expression regulation and in host cell remodeling, and it is likely that these changes reflect a physiological response of the parasite to the altered environmental conditions during culture adaptation. Similar observations were made in a comparison between five field strains and three laboratory-adapted strains [27]. We obtained independent evidence for the estimated circulation time of asexual parasites by directly estimating asexual parasite stage for each field sample, using a previously developed linear regression model [29]. Interestingly, variation in asexual parasite stage across samples correlated with variant expression patterns of individual stage-specific clusters, suggesting that parasite populations show highly synchronized cell cycle patterns during infection. These differences in parasite staging likely mask detection of expression variation in some clusters enriched in genes known to be variantly expressed.

Having developed a framework to study asexual circulation dynamics at high resolution, we applied a similar approach to define the as yet unmapped circulation and sequestration dynamics of *P. falciparum* gametocyte stages. By performing a systematic tissue screen from the autopsy of fatal malaria cases, we have recently demonstrated that immature gametocytes sequester in the hematopoietic environment of the human bone marrow before being released as mature gametocytes in the bloodstream [19]. The transition from sequestered stage IV to circulating stage V gametocyte coincides with a deformability switch from a rigid immature gametocyte to a deformable mature and transmission-competent stage V [42,68]. Due to lack of markers for the earliest gametocyte stages, we were unable to conclusively establish in our previous study whether gametocyte formation also occurs in the hematopoietic system or at another vascular site. In the latter scenario they should be found in the blood circulation prior to homing to the bone marrow.

Here we aimed to test whether these young gametocytes, similar to mature gametocytes, can be detected in peripheral blood of malaria patients. We used three complementary experiments to identify markers representing sexual commitment and the development of gametocyte rings. In particular, we identified markers by performing the first transcriptional profiling experiments during sexual commitment through isolation of a cell population enriched in sexually committed schizonts. We also identified markers that are co-expressed with *ap2-g* across patient isolates, and through further investigation of the recently published analysis of epigenetic activation of gametocytogenesis [18]. Enrichment analysis identified six putative sexual commitment clusters and five clusters enriched in gametocyte ring genes. The latter included all known young gametocyte markers and showed the same differential expression pattern across patient samples as mature gametocyte clusters. This finding was confirmed by qRT-PCR in a large cohort of cerebral malaria cases, using sentinel markers that cover development of gametocytes from the ring stage to mature, transmission-competent cells. Altogether, these data provide the first direct evidence for the quantitative presence of young gametocytes in circulation and imply that these stages originate from committed schizonts sequestered in the microvasculature. This is a very significant finding and the markers identified in the course of this study now allow targeted experiments to further investigate in tissue samples whether gametocyte formation occurs exclusively in the vasculature before the gametocyte ring homes to the bone marrow, or whether it can also take place directly in the hematopoietic system. The data generated represent a rich community resource that may also provide a basis for field diagnostic purposes and for future intervention strategies targeting the intra-erythrocytic stages of the parasite.

## Additional files

Additional file 1: Table S1. Clusters annotations. Shown are PlasmoDB accession numbers for each gene and enrichment of annotations based on q-values. Annotations are ordered in columns and defined in the corresponding header. Please refer to the Methods section for definition of all annotations. Additional file 2: Table S2. Clusters with GO function enrichments q-values. Enrichment q-values of clusters and GO functions that are <0.05 are marked in red. The ‘n’ column gives the numbers of genes in each cluster. Additional file 3: Table S7. Differential gene expression in HP1 knock-down versus wild-type parasites. Shown here are the estimated cluster-wise fixed coefficients, their standard errors, and the Benjamini-Hochberg FDR corrected q-values for the mean z-scores of the 7 to 9 hour and 10 to 12 hour coefficients. Additional file 4: Table S5. Normalized microarray data from flow sorting experiments. The microarray data were log-2 transformed. The ‘Nominal p values from t test’ column gives the *P*-value from an equal-variance two-sided *t*-test. Expression fold change of each gene was calculated as the ratio of mean of unlogged expression of the fluorescent and the non-fluorescent datasets. Genes are ranked based on their nominal *P*-values, and then ordered according to the direction of their fold changes and their *P*-values. Additional file 5: Table S6. Aggregate normalized co-expression of cluster members with *PFL1085w*. Genes are ordered according to their z-score normalized Pearson correlations with *PFL1085w* (the ‘Z (Distance)’ column). Additional file 6: Table S8. Primers with PCR efficiencies used in this study for qRT-PCR purposes. Additional file 7: Figure S1. Comparison of the functional linkage network with previously published networks. **(A)** Similarity of functional linkage (edge) weights with previously published binary networks. The Date and Stoeckert [23] and Hu et al. [24] networks include binary edge presence/absence only. We compared these to our fully connected, weighted network using a receiver operating characteristic (ROC) curve. Only genes present in each pair of networks compared were included in the evaluation. Overall genome-wide similarity is quite high despite substantial differences in integration methods and integrated data, particularly for the Date and Stoeckert network. FPR, false positive rate. TPR, true positive rate. **(B)** Similarity of clusters within networks. To determine the similarity of clusters defined within our and previous networks, we calculated the Jaccard index between all pairs of clusters in Hu *et al*. [24] to assess the overlap between their constituent gene groupings. Clusters from our network are ranked by peak time (y-axis) and those from Hu *et al*. sorted accordingly on the x-axis. We recovered clusters similar to (Jaccard >0.1) the majority of those previously defined, in addition to over 100 new clusters. Additional file 8: Table S3. Detailed annotations for individual genes within clusters with significant enrichments from Table S1. Additional file 9: Table S4. q-values for correlations between clusters and clinical parameters. The Simes (positive correlations) and Simes (negative correlations) sheets correspond to Benjamini-Hochberg FDR corrected q-values for one-sided Kruskal-Wallis tests or Fisher transformed Spearman correlation tests in the positive and negative direction, respectively. q-values ≤0.05 are marked in red. Additional file 10: Figure S2. Variant expression measurements. **(A)** Co-expression of sentinels from clusters 18, 23 and 90 by qRT-PCR (ddCT). Shown is transcript abundance for the three markers *PF14_0752*, *PF11_0512* and *PFL2565w* (cluster 23), the two markers *PFE0060w* and *PFB0095c* (cluster 90) and one marker (cluster 18) in a set of 31 samples that were collected from a cohort of cerebral malaria cases in Blantyre, Malawi. Data were normalized using the constitutive marker *seryl-tRNA synthetase*, and a baseline represented by a sample from the culture-adapted parasite strain CS2. Note that genes from both clusters are up-regulated during infection compared with CS2, an observation we also made when measuring up-regulation of all clusters based on the microarray datasets (Figure 4C; Additional file 1). **(B)** Co-expression of genes within variant groups 1 and 2 across samples. Shown are clusters 18 and 23 (variant group 1) and clusters 90 and 92 (variant group 2). Heat maps with patient samples are sorted by asexual parasite stage in each sample as plotted in Figure 5B.

## Abbreviations

FACS: fluorescence activated cell sorting
FDR: false discovery rate
GO: Gene Ontology
IDC: intra-erythrocytic developmental cycle
qRT-PCR: quantitative reverse-transcriptase polymerase chain reaction
RBC: red blood cell
