# Supplementary material for: Transcriptional profiling defines dynamics of parasite tissue sequestration during malaria infection
Source: Genome Med. 2015 Feb 27;7(1):19. doi: 10.1186/s13073-015-0133-7 (PMC4342211; doi:10.1186/s13073-015-0133-7)
Supplement: Additional file 6: Table S8. — Primers with PCR efficiencies used in this study for qRT-PCR purposes. [file 13073_2015_133_MOESM6_ESM.docx]

| **Gene Name** | **Gene Annotation** | **Other description** | **Primer Efficiency** | **Primer sequence Fw/ Rv**  **(5’ to 3’)** |  |  |
| --- | --- | --- | --- | --- | --- | --- |
| *PF14_0752* | *Plasmodium exported protein (PHISTa), unknown function* | Cluster 23/ Variant Group 1 | 100.3% | AA GTT GTT GTA GTG GTA ATT CTG A  ACA CCA AGT GTA TGA TTC CA |  |  |
| *PF11_0512* | *Ring-infected erythrocyte surface antigen 2* | Cluster 23/ Variant Group 1 | 100.8% | AGC AGA CGT ATA TGG ACG TAA TCA  CGT TAT CAA CTT CAG AAG GCA C |  |  |
| *PFL2565w* | *Plasmodium exported protein (PHISTa), unknown function* | Cluster 23/ Variant Group 1 | 109.1% | GCG TAA CTG AAC AGT TGA CAA GAG AGG AG  CAT ACG AAC ATC CTT GCC ATG TG |  |  |
| *PFB0900c* | *Plasmodium exported protein (PHISTc), unknown function* | Cluster 18/ Variant Group 2 | 97.43% | TGC TTT ATA TAA TAT CCT CAT ACA GAT  CTT AAG ATA TTC ATC ACT TCT TCA TCT |  |  |
| *PFE0060w* | *Parasite-infected erythrocyte surface protein* | Cluster 90/ Variant Group 2 | 102.4% | CCC AAA TCT GGC CAT AAG GGA CAT  TCA TCA TGA TTT GGC GTG TGT GT |  |  |
| *PFB0095c* | *Erythrocyte membrane protein 3* | Cluster 90/ Variant Group 2 | 99.5% | AGG GAT CCT AGA ACT AAG GAG GCA  TGC TTT CTG CGC GTC ATT CT |  |  |
| *PF14_0744* | *PHISTa* | Cluster 44/  Gametocyte Ring | 71.9 | ACA CGC GCA ACA ATT CTT ACA GGC  AGG CTT CTC GAC TTC CTC GAA CAT |  |  |
|  |  |  |  |  |  |  |
|  |  |  |  |  |  |  |
|  |  |  |  |  |  |  |
|  |  | | |  |  |  |
|  |  |  |  |  |  |  |
|  |  |  |  |  |  |  |
|  |  |  |  |  |  |  |
|  |  |  |  |  |  |  |
