# Supplementary figures and images for: Transcriptional profiling defines dynamics of parasite tissue sequestration during malaria infection
Source: Genome Med. 2015 Feb 27;7(1):19. doi: 10.1186/s13073-015-0133-7 (PMC4342211; doi:10.1186/s13073-015-0133-7)

**A**

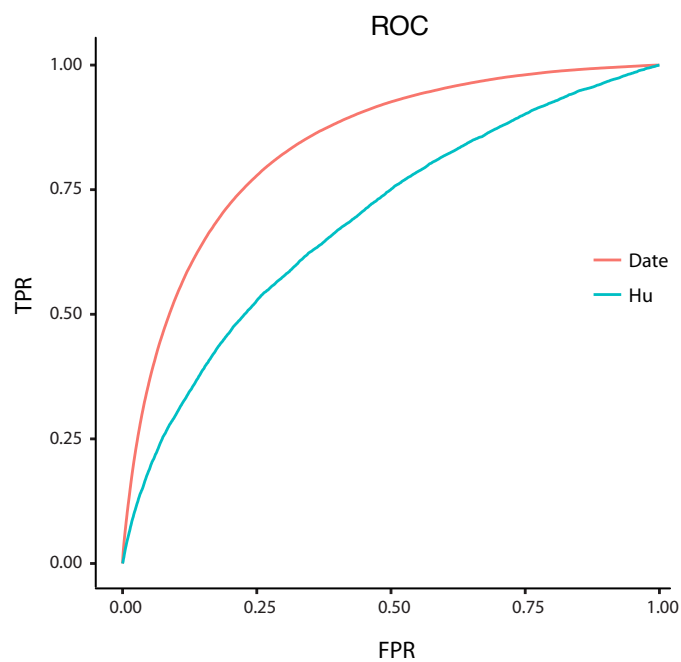

**B**

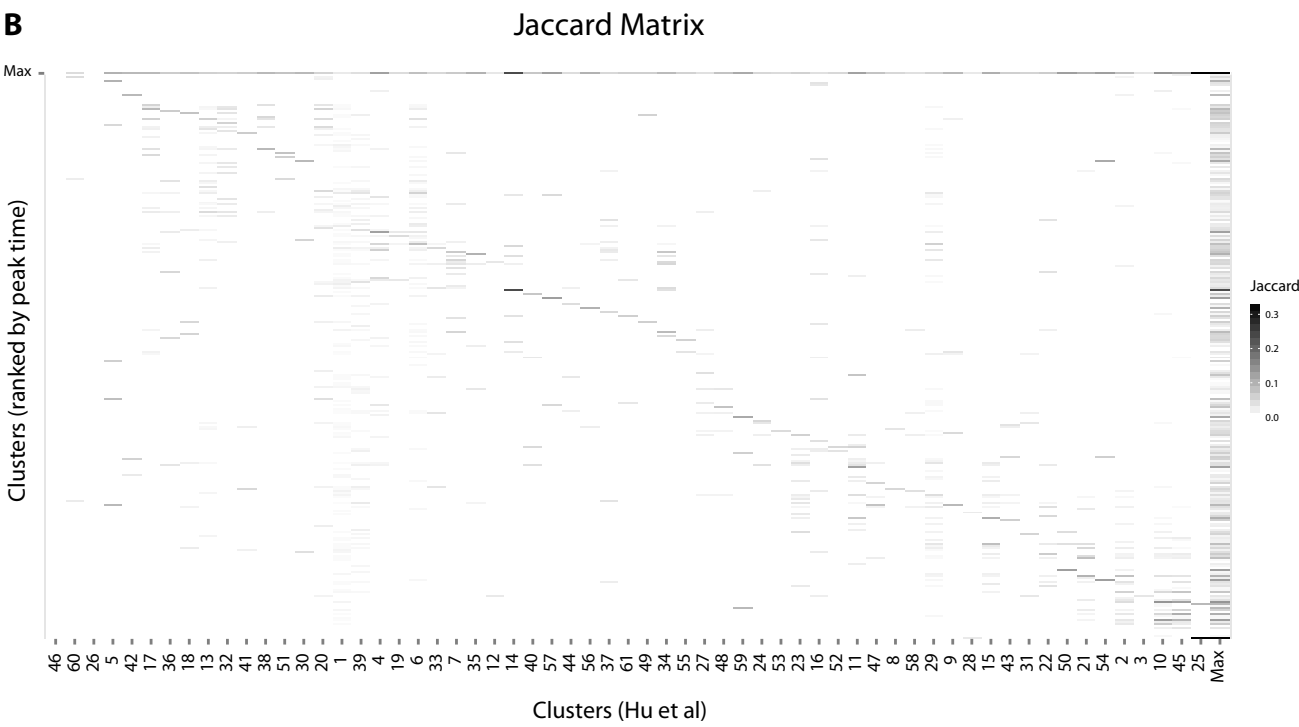

Supplement: Additional file 7: Figure S1. — Comparison of the functional linkage network with previously published networks. (A) Similarity of functional linkage (edge) weights with previously published binary networks. The Date and Stoeckert [23] and Hu et al. [24] networks include binary edge presence/absence only. We compared these to our fully connected, weighted network using a receiver operating characteristic (ROC) curve. Only genes present in each pair of networks compared were included in the evaluation. Overall genome-wide similarity is quite high despite substantial differences in integration methods and integrated data, particularly for the Date and Stoeckert network. FPR, false positive rate. TPR, true positive rate. (B) Similarity of clusters within networks. To determine the similarity of clusters defined within our and previous networks, we calculated the Jaccard index between all pairs of clusters in Hu et al. [24] to assess the overlap between their constituent gene groupings. Clusters from our network are ranked by peak time (y-axis) and those from Hu et al. sorted accordingly on the x-axis. We recovered clusters similar to (Jaccard >0.1) the majority of those previously defined, in addition to over 100 new clusters. [file 13073_2015_133_MOESM7_ESM.pdf]
