# Supplementary material for: Transcriptional profiling defines dynamics of parasite tissue sequestration during malaria infection
Source: Genome Med. 2015 Feb 27;7(1):19. doi: 10.1186/s13073-015-0133-7 (PMC4342211; doi:10.1186/s13073-015-0133-7)
Supplement: Additional file 10: Figure S2. — Variant expression measurements. (A) Co-expression of sentinels from clusters 18, 23 and 90 by qRT-PCR (ddCT). Shown is transcript abundance for the three markers PF14_0752, PF11_0512 and PFL2565w (cluster 23), the two markers PFE0060w and PFB0095c (cluster 90) and one marker (cluster 18) in a set of 31 samples that were collected from a cohort of cerebral malaria cases in Blantyre, Malawi. Data were normalized using the constitutive marker seryl-tRNA synthetase, and a baseline represented by a sample from the culture-adapted parasite strain CS2. Note that genes from both clusters are up-regulated during infection compared with CS2, an observation we also made when measuring up-regulation of all clusters based on the microarray datasets (Figure 4C; Additional file 1). (B) Co-expression of genes within variant groups 1 and 2 across samples. Shown are clusters 18 and 23 (variant group 1) and clusters 90 and 92 (variant group 2). Heat maps with patient samples are sorted by asexual parasite stage in each sample as plotted in Figure 5B. [file 13073_2015_133_MOESM10_ESM.pdf]

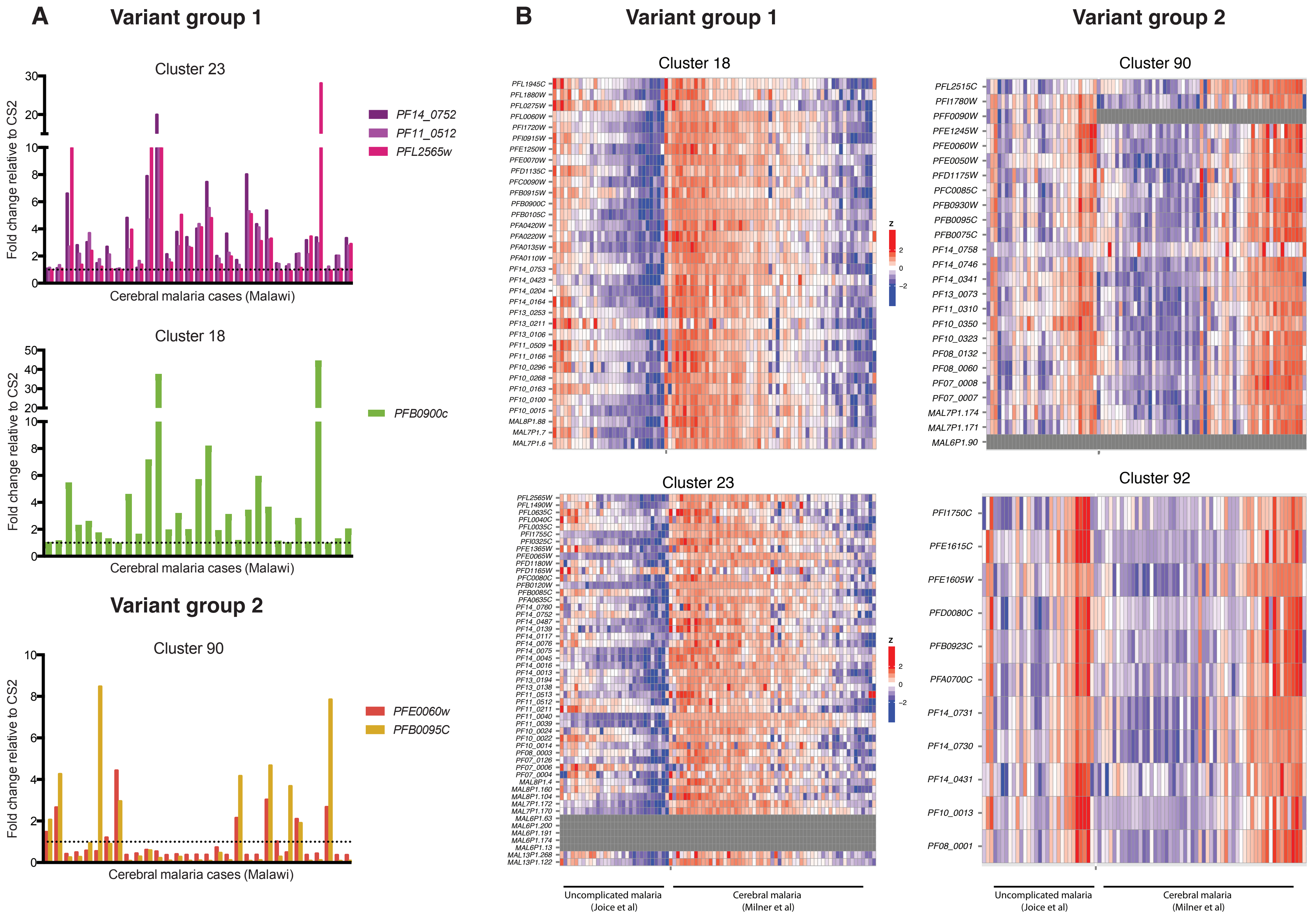
A
Variant group 1
Cluster 23
Fold change relative to CS2
PF14_0752
PF11_0512
PFL2565w
Cerebral malaria cases (Malawi)
Cluster 18
Fold change relative to CS2
PFB0900c
Cerebral malaria cases (Malawi)
Variant group 2
Cluster 90
Fold change relative to CS2
PFE0060w
PFB0095C
Cerebral malaria cases (Malawi)
B
Variant group 1
Cluster 18
PFL1945C
PFL1880W
PFL0275W
PFL0060W
PFI1720W
PFI0915W
PFE1250W
PFE0070W
PFD1135C
PFC0090W
PFB0915W
PFB0900C
PFB0105C
PFA0420W
PFA0220W
PFA0135W
PFA0110W
PF14_0753
PF14_0423
PF14_0204
PF14_0164
PF13_0253
PF13_0211
PF13_0106
PF11_0509
PF11_0166
PF10_0296
PF10_0268
PF10_0163
PF10_0100
PF10_0015
MAL8P1.88
MAL7P1.7
MAL7P1.6
Cluster 23
PFL2565W
PFL1490W
PFL0635C
PFL0040C
PFL0035C
PFI1755C
PFI0325C
PFE1365W
PFE0065W
PFD1180W
PFD1165W
PFC0080C
PFB0120W
PFB0085C
PFA0635C
PF14_0760
PF14_0752
PF14_0487
PF14_0139
PF14_0117
PF14_0076
PF14_0075
PF14_0045
PF14_0016
PF14_0013
PF13_0194
PF13_0138
PF11_0513
PF11_0512
PF11_0211
PF11_0040
PF11_0039
PF10_0024
PF10_0022
PF10_0014
PF08_0003
PF07_0126
PF07_0006
PF07_0004
MAL8P1.4
MAL8P1.160
MAL8P1.104
MAL7P1.172
MAL7P1.170
MAL6P1.63
MAL6P1.200
MAL6P1.191
MAL6P1.174
MAL6P1.13
MAL13P1.268
MAL13P1.122
Uncomplicated malaria
(Joice et al)
Cerebral malaria
(Milner et al)
Variant group 2
Cluster 90
PFL2515C
PFI1780W
PFF0090W
PFE1245W
PFE0060W
PFE0050W
PFD1175W
PFC0085C
PFB0930W
PFB0095C
PFB0075C
PF14_0758
PF14_0746
PF14_0341
PF13_0073
PF11_0310
PF10_0350
PF10_0323
PF08_0132
PF08_0060
PF07_0008
PF07_0007
MAL7P1.174
MAL7P1.171
MAL6P1.90
Cluster 92
PFI1750C
PFE1615C
PFE1605W
PFD0080C
PFB0923C
PFA0700C
PF14_0731
PF14_0730
PF14_0431
PF10_0013
PF08_0001
Uncomplicated malaria
(Joice et al)
Cerebral malaria
(Milner et al)
